# Assessing Psychiatric Morbidity and Social Support in the Transgender Community of a Coastal Indian town – A Cross-sectional Study

**DOI:** 10.1192/j.eurpsy.2026.12045

**Published:** 2026-07-31

**Authors:** B. Dahiya, V. P, S. Shetty

**Affiliations:** 1 Psychiatry, Yenepoya Medical College and Hospital; 2 Psychiatry, Father Muller Medical College and Hospital, Mangalore, India

## Abstract

**Introduction:**

Transgender individuals whose gender identity differs from their biological sex face significant discrimination, leading to a lack of access to essential resources such as education, employment, housing, and healthcare. The study focuses on assessing psychiatric morbidity and social support in this marginalized group to aid in better healthcare and societal integration.

**Objectives:**

1. To evaluate the presence of psychiatric diagnoses in the transgender community.

2. To assess patterns of social support within the community and establish clinical correlates.

**Methods:**

This observational, cross-sectional study used Snowball sampling to recruit 94 participants who were above 18 years, identified as transgender (man or woman), and consented to participate. Assessment tools like MINI Plus for psychiatric diagnoses, the Social Support Rating Scale (SSRS) for measuring social support, and the Self-Injurious Thoughts and Behaviours Interview-Short Form (SITBI-SF) for assessing self-harm behaviors were used.

**Results:**

Majority (69.1%) had not completed basic education; 77.6% were engaged in begging or commercial sex work, and most were single (80.9%). Psychiatric comorbidity was present in 54.3%, with substance dependence (27.6%) and major depressive disorder (12.8%) among the most common. Suicidal ideation lifetime prevalence was 36.2%, with 11.7% having attempted suicide. Social support scores were generally low; subjective support was higher among those with more education and those in relationships.

**Table 1.** Psychiatric comorbity (n=94) Table 1. Long description.

Number of Psychiatric comorbidities	Frequency	Percentage
None	43	45.7
1	35	37.2
2	7	7.5
3	9	9.6

**Table 2.** Comparison of Social support rating score and Psychiatric comorbidities using independent sample t-test (*p value <0.05 is considered to be statistically significant) Table 2. Long description.

Domains of Social Support	Current Psychiatric comorbidities present (n=38)	Current Psychiatric comorbidities absent (n=56)	Test statistics	p value
Objective support	5.76±0.883	6.48±1.27	-3.010	0.003*
Subjective support	18.11±3.22	18.82±2.58	-1.192	0.236
Use of support	9.13±1.58	9.23±1.41	-0.323	0.748

**Conclusions:**

Despite a huge step like the Indian government enacting laws recognizing transgender rights, psychological and physical health needs remain largely neglected in smaller towns. This study highlights the transgender community’s challenges which lead to high rates of psychiatric morbidity and suicidality. Early screening is essential and social support, especially from employment and relationships, plays a protective role. These findings can inform prevention and intervention strategies to improve mental health outcomes and social reintegration for transgender individuals in similar settings.

**Disclosure of Interest:**

None Declared

## Long descriptions

### Table 1. Long description

The table consists of three columns: Number of Psychiatric comorbidities, Frequency, and Percentage.

* Row 1: None, Frequency 43, Percentage 45.7.

* Row 2: 1, Frequency 35, Percentage 37.2.

* Row 3: 2, Frequency 7, Percentage 7.5.

* Row 4: 3, Frequency 9, Percentage 9.6.

Navigate back to Table 1..

### Table 2. Long description

The table consists of five columns: Domains of Social Support, Current Psychiatric comorbidities present (n equals 38), Current Psychiatric comorbidities absent (n equals 56), Test statistics, and p value.

* Objective support: Present group mean is 5.76 plus or minus 0.883; Absent group mean is 6.48 plus or minus 1.27; Test statistic is -3.010; p value is 0.003 (statistically significant).

* Subjective support: Present group mean is 18.11 plus or minus 3.22; Absent group mean is 18.82 plus or minus 2.58; Test statistic is -1.192; p value is 0.236.

* Use of support: Present group mean is 9.13 plus or minus 1.58; Absent group mean is 9.23 plus or minus 1.41; Test statistic is -0.323; p value is 0.748.

Navigate back to Table 2..

